# Environmental Psychology Effects on Mental Health Job Satisfaction and Personal Well Being of Nurses

**Published:** 2015-06

**Authors:** Sodeh Tavakkoli, Mohammad Mahdy Asaadi, Amir H Pakpour, Marzieh Hajiaghababaei

**Affiliations:** 1 Landscape Architecht, Member of IFLA ( International Federation of Landscape Architects); 2 Department of Psychology , University of Payam-e –Noor, Tehran, Iran; 3 Social Determinants of Health Research Center, Qazvin University of Medical Sciences,Qazvin, Iran.; 4 Brain and Spinal Injury Research Center, Neuroscience Institute, Tehran University of Medical Sciences, Tehran, Iran

**Keywords:** *Environmental Psychology*, *Mental Health*, *Job Satisfaction*, *Well-being*, *Nurses*

## Abstract

**Objective:** Environmental psychology as a science could be useful in understanding the dissociation between the man and the environment. The aim of this study was to compare mental health, job satisfaction and well-being of nurses who work in hospital environments with different designs.

**Material:** This was a quasi-experimental study, in which 250 nurses filled out the mental health, well-being and job satisfaction questionnaires. They were categorized into 3 groups randomly. Group1 included 63 nurses who worked in an environment without any natural elements; group 2 included 100 nurses who worked in an environment with natural elements and group 3 included 87 nurses who worked in an environment without any psychological and ergonomic design. The last group was only stimulated by demonstrating visual stimulus. Data were analyzed using the ANOVA and Tukey’s pursuit statistical method.

**Results:** The nurses who were working in an environment without any natural elements reported significantly lower scores on mental health, well-being and job satisfaction compared to those who were working in other groups, with the exception of social functioning.

Moreover, depression and anxiety were more common in nurses who were working in environments without any natural elements compared to those in the other groups (p<0.05).

**Conclusions:** We can increase job satisfaction, and mental health and well-being of the nurses through the use of natural design and environmental psychology indexes in hospital buildings.

Today, Urbanization and separation of humans from nature have caused enormous damages to human beings. Influx to the cities and lack of space caused the loss of natural spaces which is one of the most important stressful factors for humans (1). Researches have shown that lack of each person’s control over the environment and existence of problems in design-engineering system of the buildings caused congestion and social isolation which ultimately have led to some problems regarding job and social features; and consequently, these factors endanger the mental health of the individuals (2).According to the World Health Organization (WHO), the full ability to take social, psychological and physical roles is called health. Patient’s environment can play a crucial role in improving the therapeutic efficacy as well as patient-reported outcomes (3). Environmental psychology as a science could be useful in understanding the dissociation between the man and the environment (4), and it indicates that how location specification affect behavior and helps the behavior change and causes compatibility with the environment (5).

In broken window theory, Wilson et al. (1982) showed that environment has powerful impacts on human health and wellbeing (6). Personal wellbeing shows individuals’ satisfaction and happiness about their life quality and is a positive status of physical, psychological and social welfare (7-8). One of the aspects of environment that may affect human health is Building Environment (BE). Conceptually, Building Environment includes all of constructed environments in which humans live and work (9), and it also includes building design and interior architecture and effects human’s emotions, excitement and performance (10). 

Nursing is one of the stressful professions. Working pressure, by itself, is considered a source of frustration for nurses. Nurses are the man power inside the hospitals and are the main service providers in the health care system and they significantly affect the health and hygiene level of the society by providing various health related services to the patients (11). Nurses are always exposed to the damages caused by stress, working environment and activities related to their job (12). Gelman and Turner in an article about nurses’ quality of life have found that hospital environment directly affects individual's quality of life (13).

One of the issues raised in nursing services management is the low level or lack of job satisfaction. Reduced attention quality, leaving work and absence from work are some of the negative effects of low job satisfaction. (14). Lack of job satisfaction is one of the important factors in nurses quitting their job (15). Job satisfaction is an underlying attitude to create tendency, interest, talent and preparation in order to give proper response to the working environment in personal and social features and includes the features and requirements of a job with external environment and interpersonal relations with working situation (16). Psychologists also believe that environment ergonomics and design may reduce physical and mental stressors and pressures inside the working environment (17). 

Using the environmental psychology principles in location designs (especially due to the treatment of environment which is discussed in the holistic medicine) promotes mental health and wellbeing (18). Watching the nature from a window increases the sense of wellbeing (19); also, using natural elements in the work environment results in the following positive outcomes: Reducing the environmental stress and increasing calmness (18), less depression and more mental compatibility (20), positive effects on happiness and quality of life (21), acting as a buffer against the stressful events (22), improving individual health (23), increasing the compatibility, having a sense of safety, experiencing positive emotion, reducing angriness and increasing patience (24). 

Therefore, this research compares mental health, job satisfaction and wellbeing of nurses depending on their working environment. 

The hypothesis of this research was that the indexes of job satisfaction, wellbeing, mental health and their subscales (somatic symptoms, anxiety and insomnia, social dysfunction and severe depression) were different in the three groups of the nurses who worked in three different work environments: the nurses who worked in an environment with no interior design (1), nurses who worked in an environment with natural beautiful perspective, (2) and those nurses who worked in an environment with no interior design, but with limited view of the simulated garden (3).

## Material and Methods

This was a quasi-experimental study. The sample included 250 male and female nurses with the age range of 23- 47 years who were randomly selected from Khatamol-Al-Anbia hospital located in the third district of Tehran municipality.

 This sample was substituted stochastically in 3 groups randomly. The first group of the nurses (n = 63) worked in the basement of the hospital where the design was not in accordance with the psychological-environmental principles, and the nurses who worked there experienced an ordinary and cold environment and they had no access to the designed environment; the second group ( n = 100) worked in a small garden simulated area which had been designed based on psychological principles (25, 26) and the third group ( n= 87) worked in an area, from which they could see the outside designed environment through a distant window. The sampling has been done in two shifts of morning and afternoon. All participants were asked to complete the anxiety and depression questionnaire at baseline. 

Job situation and working volume and salary were equal in all the participants, and we tried to remove all the interruptive factors. 

 Sample size was calculated based on a previous study. A hypothesized mean which was considered for this study was 4.96 for job satisfaction in the control group (SD51.09). Power-calculations to detect small effects (12%) on scores of job satisfaction changes with a power of 0.80 (p < 0.05) resulted in a target sample size of 50 for each group (27)

This garden simulated location was designed based on environmental psychology using known natural elements in an environment covered by plants and green elements with a combination of jungle outlook and canebrake with a short waterfall and gentle sound of the birds; also, there were some comfortable chairs made of bamboo wood for the resting time of the nurses below a canopy (Fig 1). The garden simulated location could be seen from all parts of this section; and when windows were opened, the nurses could hear the sound of the waterfall and birds during their work.

During the break time, the nurses could use the resting area inside the garden simulated area. At the same floor of the hospital after leaving this section at the end of opposite corridor, there was an area where the nurses could only look at the garden simulated location from a far distance and there was no access to the garden (Fig 2). At the same hospital, there were some areas in the basement floor where there were no environmental factors with a in a quiet dimly lit room even there were no windows. In other words, the hard and cold environment of the hospital cannot be seen well enough.

General Health Questionnaire (GHQ): The questionnaire of general health arbitrated to Goldberg& Hillier for screening the non-psychotic mental disorders has been designed with 28 questions on a 4- point Likert- scale and 4 subscales about somatic symptoms, anxiety and insomnia, social dysfunction and severe depression (29,28). This tool has been translated into Persian and was found to be highly valid and reliable (30).

Job Satisfaction Index (JSI): The Job Satisfaction Index (JSI) which has been made by Smith and Kendal (1996) is a scale with 20 items which is scored on a 5-point Likert scale. Smith and Kendal believe that this scale has high reliability and validity. They have reported the reliability coefficient of the sub tests in the first study from 59% to 93% and in the second study from 62% to 93%. (31) Haghayegh Khorasani reported the reliability coefficients of the subtests as 0.59-0.92 in the first study and 0.62-0.93 in the second study in Iran (32). In the present study, reliability of JSI was obtained 83% by Cronbach's α and content validity was approved by the faculty. 

Personal wellbeing questionnaire: The personal wellbeing questionnaire of adults named “Personal Wellbeing Index” has been standardized in 2002. The current scale has 8 items, each of which asks questions about one field on a 7- point likert- scale. Aghayousefi (2004) has found the reliability of the research to be 0.84 and in another research by using Cronbach's α; also, he has found the reliability of the test equal to 0.87 (33). 

The current research has been performed three months after designing the hospital environment. Then, some nurses used that simulated garden, some looked at the simulated garden from a far distance and the others were located in the basement with no environmental psychology. In this research, the indexes of mental health, job satisfaction and wellbeing of the nurses working in this hospital have been compared considering the equality of all working situations and only based on the differences between working environment indexes. The findings were entered to SPSS- 17 and have been analyzed by Variance Analysis method and then by Tukey’s pursuit method.

## Results

The study participants consist of were 250 nurses, (62 men and 188 women); Of whom, 100 were married and 150 were single. All participants had tertiary education. There was not any significant difference between the groups in terms of anxiety and depression at baseline (P>0.05). We used variance analysis to The findings related to compare and survey the differences among the means of the three groups in the general health index, job satisfaction and wellbeing. 

As depicted in Table 1, significant differences between the three groups were found. Nurses who were working in an environment with interior design reported significantly higher general health compared than those working in an environment with no interior design (Table 1). Considering the current results, at significance level of p<0.05, the general health of the nurses working in a garden simulated area and the nurses who only viewed the environment from the window was different from the nurses working in an environment with no design. Moreover, considering the results of the Tukey test, it can be stated that at the same level of significance, the general health of the nurses working in a garden simulated environment and the nurses who looked at the environment from a window was better than the nurses who are worked in an area without any designs. 

As demonstrated in Table 2, the comparison among the sub scales of the general health shows that the anxiety of those nurses working in the garden simulated area and the nurses who looked at the garden simulated area from a window were significantly less than the nurses working in the area without any designs (p<0.05). On the other hand, depression of the nurses working in the garden simulated setting and the nurses looking at the garden simulated location from a window was less than the first group. No significant difference was found between the three groups in respect to the social function subscale. 

As shown in table 3, the difference among the three groups was statistically significant on job satisfaction, and the mean of job satisfaction of the second group is was higher than the third and the first groups; also, the mean of job satisfaction of the second group was higher than the third group. 

 Considering the results of variance analysis and Tukey pursuit test, it can be said that the job satisfaction of in the second group (those working in a simulated area) was higher than of the third group (those who were working in an area having access to the designed area via a window) at significance level of 0.01. Furthermore, considering Tukey’s analysis, it can be said that job satisfaction level of the nurses working in a garden simulated area and nurses working in the area with windows was significantly more than those nurses working in the area with no design.

As shown in Table 4, the mean of wellbeing index on the nurses working in a garden simulated area was higher than the mean of wellbeing index of the nurses working in the area without design with a small difference comparing compared to the nurses looking at the designed area from a window. 

**Table1:** Descriptive Statistics and Analysis of Variance and the Tukey Test on the General Health of the Nurses in Different Working Environments

**General ** **Health**	**n**	**Mean**	**SD**	**F**	**Mean Differences ** **(Tukey)**	**SD error**	***P *** **- value**
Group1	63	21.21	4.94	4.4	(1&2) 4.408	1.02	0.011
Group2	100	33.61	3.64		(1&3)1.372	1.06	0.019
Group3	87	23.29	5.367		(2&3) 0.525	2.70	0.807

group1: Nurses working in an environment with no interior design

group2: Nurses working in an environment with a natural beautiful outlook

group3: Nurses working in an environment with no interior design, but with limited view of the simulated garden

**Table 2:** Descriptive Statistics and Analysis of variance and the Tukey test on the General Health Subscales of the Nurses in Different Working Environments

**Subscales** **Of General ** **Health**	**Group**	**n**	**Mean**	**SD**	**F**	**Mean Differences ** **(Tukey)**	**SD error**	***P *** **-value**
	1	63	8.71	1.01	5.05	1&2 0.059	0.749	0/063
Somatic symptoms	2	100	7.06	1.09		1&3 1.85	0.167	0.012
	3	87	7.97	0.30		2&3 0.406	0.596	0.568
	1	63	7.57	2.63	4.023	(1&2) 1.152	0.706	0.013
Anxiety	2	100	6.43	3.68		(1&3) 1.272	0.917	0.011
	3	87	6.31	3.03		(2&3) 0.128	0.628	0.875
	1	63	3.08	2.09	5.343	(1&2) 0.123	0.509	0.014
Depression	2	100	2.17	2.85		(1&3) 0.128	0.781	0.011
	3	87	1.93	1.39		(2&3) 0.175	0.564	0.875

group1: Nurses working in an environment with no interior design

group2: Nurses working in an environment with a natural beautiful outlook

group3: Nurses working in an environment with no interior design, but with limited view of the simulated garden

**Table 3:** Descriptive Statistics, Analysis of Variance and the Tukey Test on the Job Satisfaction of the Nurses in Different Working Environments

**Job Satisfaction**	n	Mean	SD	F	Mean Differences (Tukey)	SD error	*P *- value
Group 1	63	21.6	1.01	3.565	(1&2) 4.91	1.12	0.01
Group 2	100	35.3	1.89		(1&3) 1.35	1.05	0.14
Group 3	87	28.13	1.01		)2&3) 3.13	2.13	0.05

group1: Nurses working in an environment with no interior design

group2: Nurses working in an environment with a natural beautiful outlook

group3: Nurses working in an environment with no interior design, but with limited view of the simulated garden

**Table 4:** Descriptive Statistics and Analysis of Variance and the Tukey Test on the Wellbeing of Nurses in Different Working Environments

**Wellbeing**	**n**	**Mean**	**SD**	**F**	**Mean ** **Differences ** **(Tukey)**	**SD error**	***P *** **- value**
Group1	63	59.01	10.17	5.097	(1&2) 8.07	3.57	0.014
Group 2	100	68.98	6.69		(1&3) 6.05	2.19	0.032
Group 3	87	66.07	9.23		(2&3) 0.51	5.07	0.702

group1: Nurses working in an environment with no interior design

group2: Nurses working in an environment with a natural beautiful outlook

group3: Nurses working in an environment with no interior design, but with limited view of the simulated garden

**Figure:**
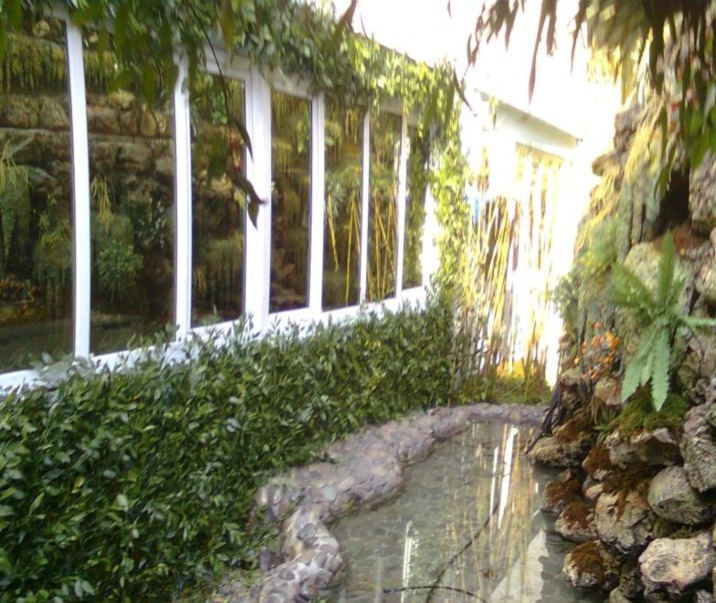


**Figure:**
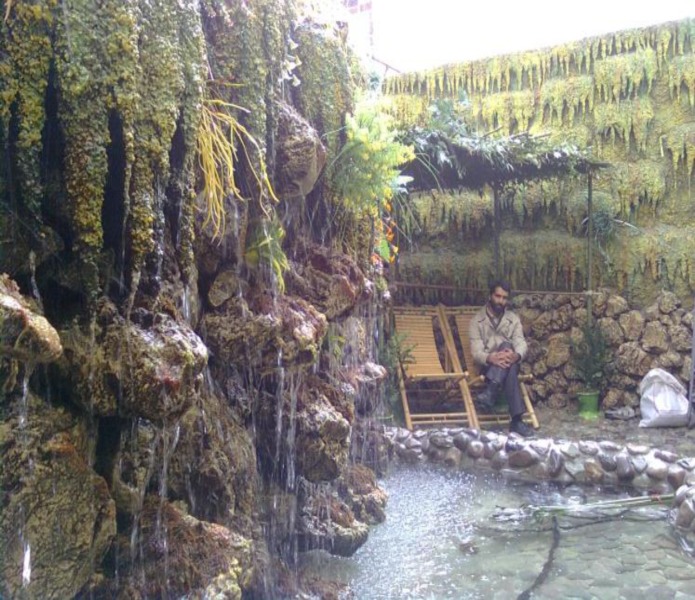


Considering the current results, the wellbeing of the nurses working in a garden simulated area was different from that of the nurses who looked at the area from a window (p<0.05). Moreover, considering Tukey’s pursuit test, it can be stated that at the same significance level, the wellbeing of the nurses working in the designed area was higher than that of the nurses who worked in the area with no design with a small difference compared to those looking at the area from a window. There was no any significant differences between nurses who were working in the garden simulated area and those looking at the area from a window.

## Discussion

The analysis of this research data revealed that the mental health of the nurses working in the garden simulated area and those working in the area with a window to a designed area was better than those working in the area with no design. Also, the results revealed a difference between the stress of the nurses working in a no- design area and the nurses working in the designed area and those looking at the designed area from a window. This result is in accordance with that of previous findings (20, 24).

We usually live in a place without realizing its effects on ourselves. Development of the cities and their extra ordinary growth had forced the architects to build the buildings in a vertical direction instead of horizontal and there is no exception about the hospitals; and this has led to the vertical growth of the hospitals and also caused a distance from the nature. 

This research revealed a difference between depressions of the nurses working in the area with no design and those working in the designed area or those looking at the area from a window; the findings were in line with researches by Perrins et al. and Evans (21, 18). Also, there was no difference between the social performances of the nurses working in the designed area, those working in the area with a window to the designed area and also those working in the area with no design. 

The findings of this research showed that this simulated garden area improved the job satisfaction and the marks of job satisfaction of the nurses working at the designed area and those with a view to the designed area. These findings are in accordance with the findings by Walsh and Knott, Moos and Insel, Hoseini and Mirzabeigi (16, 17, 14, 15). 

The other findings of this research showed that the designed area for the resting time of the nurses affected their wellbeing and that the marks of nurses’ wellbeing were higher in the group working in the designed area and those working in the area with a window in comparison with those working in the area with no natural environment. These findings are in accordance with the previous findings that showed being at a designed area reduces stress or mental fatigue (psychologically), and improves physical health and develops wellbeing (increasing social interactions and reducing panic) (13, 17,11). Also, these findings support Park’s research that showed principles of environment design affect stress and well-being (34).

Green areas in cities have been rescued significantly. On the other hands, the numbers of buildings have been increased considerably in cities. Therefore, designers and architects should closely follow the environmental psychologists in order to improve the human environment. Applying basic changes in the environment based on environmental psychology indexes, positively affects the health of the nurses. This study revealed that wellbeing, job satisfaction and mental health were significantly higher in the nurses who were working in the area with no design but watching the designed area from a window are not less than those working in the area designed with natural factors. This study indicated that places which were designed by natural elements increase worker’s mental health, job satisfaction as well as their wellbeing. 

The studies conducted on the effects of different environments on different psychological indexes are rare and it is suggested that the survey on other variables such as nurses’ quality of life with different intervals after the design be conducted in the future studies. The main limitation of this study was the lack of a proper hospital design based on psychological principles; consequently, we could not conduct an experimental study.

## Limitations

This study has a limitation as follow: the study sample had been selected from only one hospital, thus, it limited the generalizability of the study results. 

## Conclusion

By applying the environmental indexes in buildings, we can reduce the coefficient of the hardness and increase the environment psychological indexes. Because nursing is one of the most important positions in the society and the health of the nurses affects the general health of the patients, we should improve their mental health, job satisfaction and wellbeing through the use of environmental psychology.
